# An Unprecedented High Incidence of Leptospirosis in Futuna, South Pacific, 2004 – 2014, Evidenced by Retrospective Analysis of Surveillance Data

**DOI:** 10.1371/journal.pone.0142063

**Published:** 2015-11-03

**Authors:** Denis Massenet, Jean-François Yvon, Clément Couteaux, Cyrille Goarant

**Affiliations:** 1 Agence de Santé des îles Wallis & Futuna, Laboratoire de biologie médicale/hôpital de SIA, BP 4G, 98 600 Mata'Utu, Wallis & Futuna; 2 Laboratoire de Ducos, BP 3931, 98846 Nouméa, New Caledonia; 3 Institut Pasteur in New Caledonia, Institut Pasteur International Network, Leptospirosis Research and Expertise Unit, 9–11 Avenue Paul Doumer, BP 61, 98 845 Noumea, New Caledonia; Bharathidasan University, INDIA

## Abstract

Futuna is a small Polynesian island in the South Pacific with a population of 3,612 in 2013. The first human leptospirosis case was confirmed in 1997. Active surveillance started in 2004. Cases were confirmed by PCR or real time PCR, or by serology using MAT or a combination of IgM-ELISA and MAT. A retrospective analysis of surveillance data shows that the disease was endemic with a mean annual incidence of 844 cases per 100,000 over an 11-year period from 2004 to 2014. An epidemic peak as high as 1,945 cases per 100,000 occurred in 2008. Serogroup Australis was predominant until 2007, Icterohaemorrhagiae was dominant afterwards. Cluster analysis revealed different hot spots over time. Lifestyle habits, such as walking barefoot in irrigated taro fields or pig pens probably contributed to contamination from the swine and rodent reservoirs to humans. Severe forms were rare, and the case fatality rate was 0.5%. The medical community and general population were aware of leptospirosis and rapid treatment with amoxycillin was the main treatment, probably contributing to this low fatality rate.

## Introduction

Leptospirosis is a bacterial zoonosis of worldwide significance. Rodents are the main animal reservoir, but other mammals may also maintain other co-adapted pathogenic *Leptospira* strains. Transmission to humans occurs through contact of abraded skin or mucous membranes with urine of infected animals, mostly chronic asymptomatic reservoirs, either directly or via surface waters or wet soils [1]. After an incubation time of 2 to 21 days, the acute phase is usually sudden and characterized by fever, headache and myalgia. Later symptoms may include conjunctival injection, abdominal pain, vomiting, prostration, icterus, anuria or oliguria, cardiac arrhythmia or deficiency, meningeal syndrome and a skin rash. The diversity of clinical presentations is a major diagnostic challenge: the classical form known as “Weil’s disease”, with renal and hepatic involvement and hemorrhages, usually accounts for less than one third of cases. Biological confirmation is therefore needed for a relevant surveillance.

Despite being ubiquitous, leptospirosis mostly affects tropical and subtropical rural or suburban areas and high incidence rates have been reported mostly in these regions [2, 3]. Futuna is a small island in the South Pacific (14° 17′ S, 178° 07′ W), Northeast of Fiji, with a population of 3,612 in 2013. The first case of leptospirosis from Futuna was confirmed in 1997 with 1 to 11 cases a year until 2003. Active surveillance was set up in 2004. Since then, leptospirosis was systematically considered as a possible etiology for any acute fever and biological confirmation was systematically requested. Systematic information forms with demographic and geographic data as well as symptoms were also systematically requested and filled by the physician.

This retrospective study aims at describing some epidemiological features of leptospirosis in Futuna, using anonymized data from the active surveillance, to improve our understanding of leptospirosis epidemiology to better address prevention.

## Materials and Methods

### Futuna Island

Futuna is a small (64 sq. km), mountainous (Mount Puke at 524 m) tropical island in the South Pacific. The 3,612 inhabitants (2013 census) live in 14 villages along the coast. The population is declining (4,873 inhabitants at the earlier 2003 census) mostly due to emigration of young active adults. The climate is hot and humid, with high rainfall totaling 3,000 to 4,000 millimeters a year (Data kindly provided by Meteo France). Futuna (together with the uninhabited island of Alofi and the island of Wallis) belongs to the French collectivity “Wallis and Futuna”. Futuna is characterized by its geographical isolation, with a single one-hour flight from Wallis weekly. The lifestyle is traditional: pig farming and irrigated taro cultivation are the main means of subsistence and most people walk barefoot.

### Surveillance design, laboratory techniques and case definitions

Our study used retrospective surveillance data for the period 2004–2014. As part of the surveillance activity, the local health authority (“Agence de Santé de Wallis & Futuna”) collected data from both confirmed and probable cases using the notification forms filled in by medical doctors at the time of initial medical contact. This included weekly phone calls to the medical staff especially dedicated to exchange information on leptospirosis. Forms included socio-demographic and exposure data as recommended by WHO [4]. Laboratory analysis used sera collected at the first medical visit. From 2004 to 2007, all sera were analyzed using the Micro Agglutination Technique at Institut Pasteur in New Caledonia [5] using the panel shown in S1 File. From 2008 on, all sera were first analyzed using the Panbio Leptospira IgM ELISA (Alere, Australia) and ELISA-positive specimens were then sent out for MAT confirmation and possible identification of the presumptive serogroup. Additionally, acute sera were analyzed with the PCR or a real-time PCR used for diagnosis and surveillance at Institut Pasteur in New Caledonia and which changed over time to follow technological evolutions ([6] in 2004, [7] until 2011 and [8] since 2012). Patients were asked to return to give a convalescent serum sample but did not always comply. Confirmed cases were patients with current or recent fever (within a week before presentation), a positive (real-time) PCR, a seroconversion (from nil to >400 in MAT) or a significant rise in serological titers (a 2-fold increase in IgM-ELISA or a 4-fold increase in MAT titers between acute and convalescent sera). Probable cases were defined as patients with a recent (one week) or current fever and a single IgM ELISA above 15 units (higher than the 11 units recommended by the manufacturer) or a single MAT titer of at least 800 for a pathogenic serogroup. The putative infecting serogroup was identified as the serogroup with the highest titer in MAT or from the sequence polymorphism of a *lfb1* PCR product [9]. For cases diagnosed with serology and with co-agglutinins in the MAT, the infecting serogroup was considered as unknown.

### Study Type, data collection and analysis

The study was a retrospective analysis using surveillance data. The 2004–2014 surveillance file was anonymized by removing first and last names and converting the birthdate to an age class. Meteorological data were kindly provided by Météo France. The association between rainfall and leptospirosis cases was analyzed using a simple linear regression using the open source platform R; results are expressed as the coefficient of determination (R²). Census data from 2003 (4,873 inhabitants), 2008 (4,217 inhabitants) and 2013 (3,612 inhabitants) were kindly provided by the “Délégation de Futuna de l’Administration Supérieure” and used for estimating the annual island population along the duration of the study, assuming a linear decrease in population between two consecutive census. At the district level, the census 2008 data was used as the reference data. The incidence was calculated at the district level (based on 2008 reference census) and at the island level (with census values and population estiamtes inbetween). Additionally, the incidence was also calculated with only confirmed cases, in order to provide a conservative minimal value of the incidence. Fisher exact test was used to compare the contribution of serogroups between different periods. Mapping was done using the software MapInfo (v8.5). Possible spatial clusters were investigated over the study period using the freeware SaTScan (v9.3.1, Inc. http://www.satscan.org/) [10]. Briefly, the cases are each categorized by the date (here, the year) and district of occurrence (positioned at the centroid of the district), they are assumed to follow a Poisson distribution. SatScan uses a moving window (here circular) with a size equal to 50% of the population studied. Using 1000 Monte Carlo permutations, SatScan identifies clusters as non-random distribution of cases. A p value ≤ 0.05 was considered as statistically significant for all analyses.

### Ethics Statement

All patients were informed of the surveillance program for leptospirosis and that the data included in their notification files could be used for surveillance purpose after anonymization. As per regulations currently applicable in Wallis and Futuna, no specific ethical approval was required for the study presented here.

## Results

**Fig 1:**
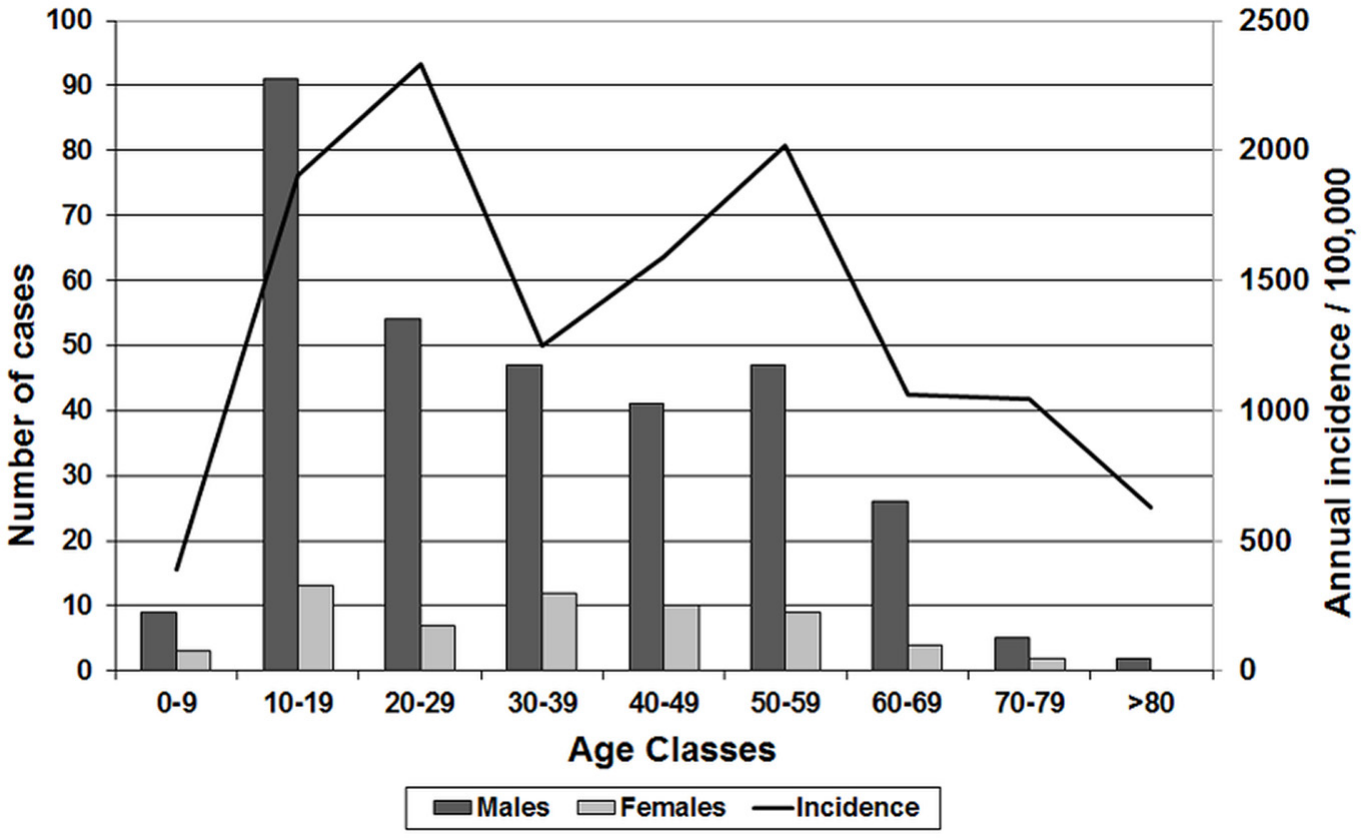
Number of cases of leptospirosis during 2004–2014 by age class and gender and average age specific annual incidence during 2007–2009 (age structure of 2008 census data used).

Both confirmed and probable cases were analysed together as “leptospirosis cases”. Most leptospirosis cases were males (M/F = 5.4), with a peak incidence in the 20–29 y.o. age class (Fig 1). The clinical presentation was most frequently classical, with fever, headache and myalgia in 98%, 84% and 82% of patients respectively. Only 11% of the patients initially presented with an icterus. They presented on average 2 days after symptom onset and were treated with intravenous amoxycillin. Only two fatalities were recorded, a 0.5% fatality rate.

**Fig 2:**
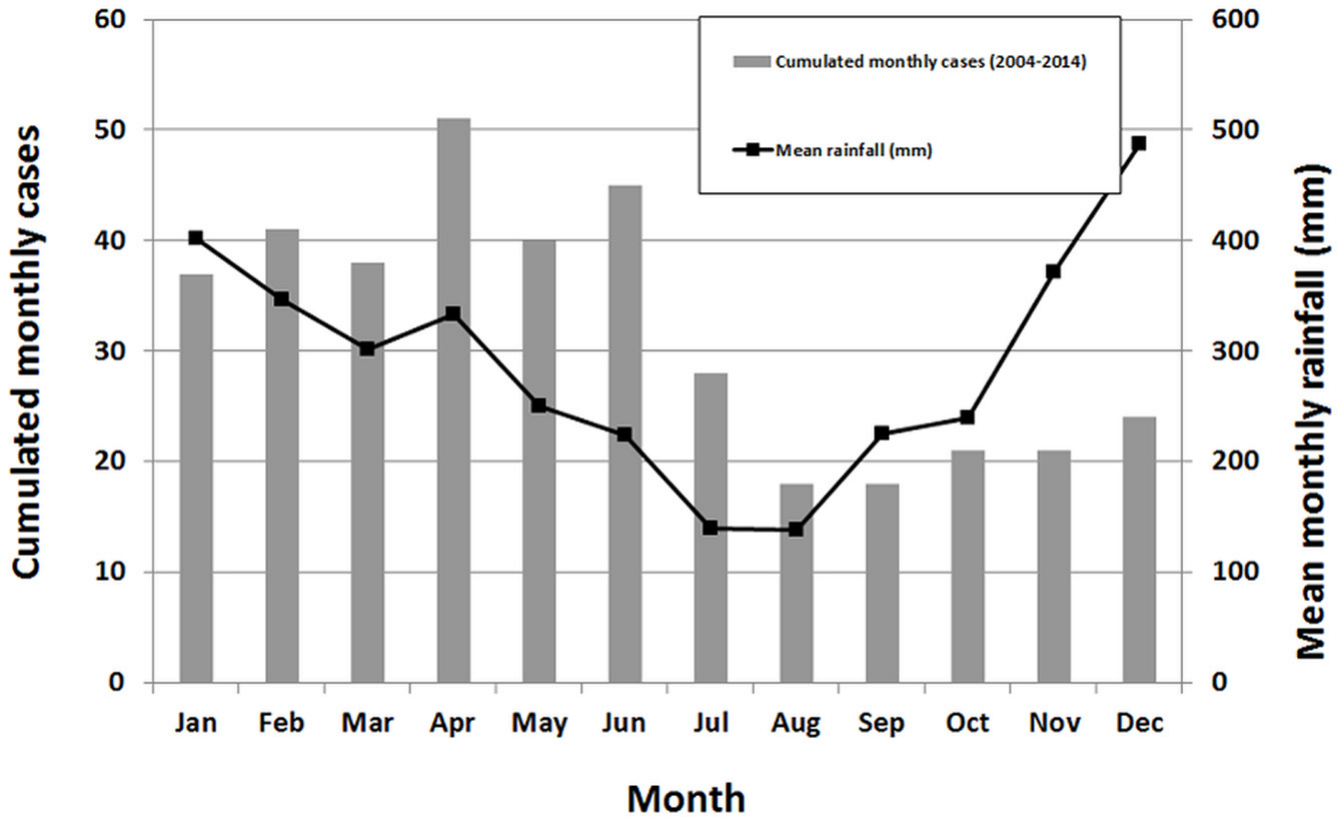
Cumulated monthly cases of leptospirosis and average monthly rainfall in Futuna, 2004–2014.

The disease was seasonal, with a majority of cases (66%) during the first half-year, as shown in Fig 2, and was correlated with the rainfall observed two months earlier (R² = 0.569).

### Dynamics over the study period

**Fig 3:**
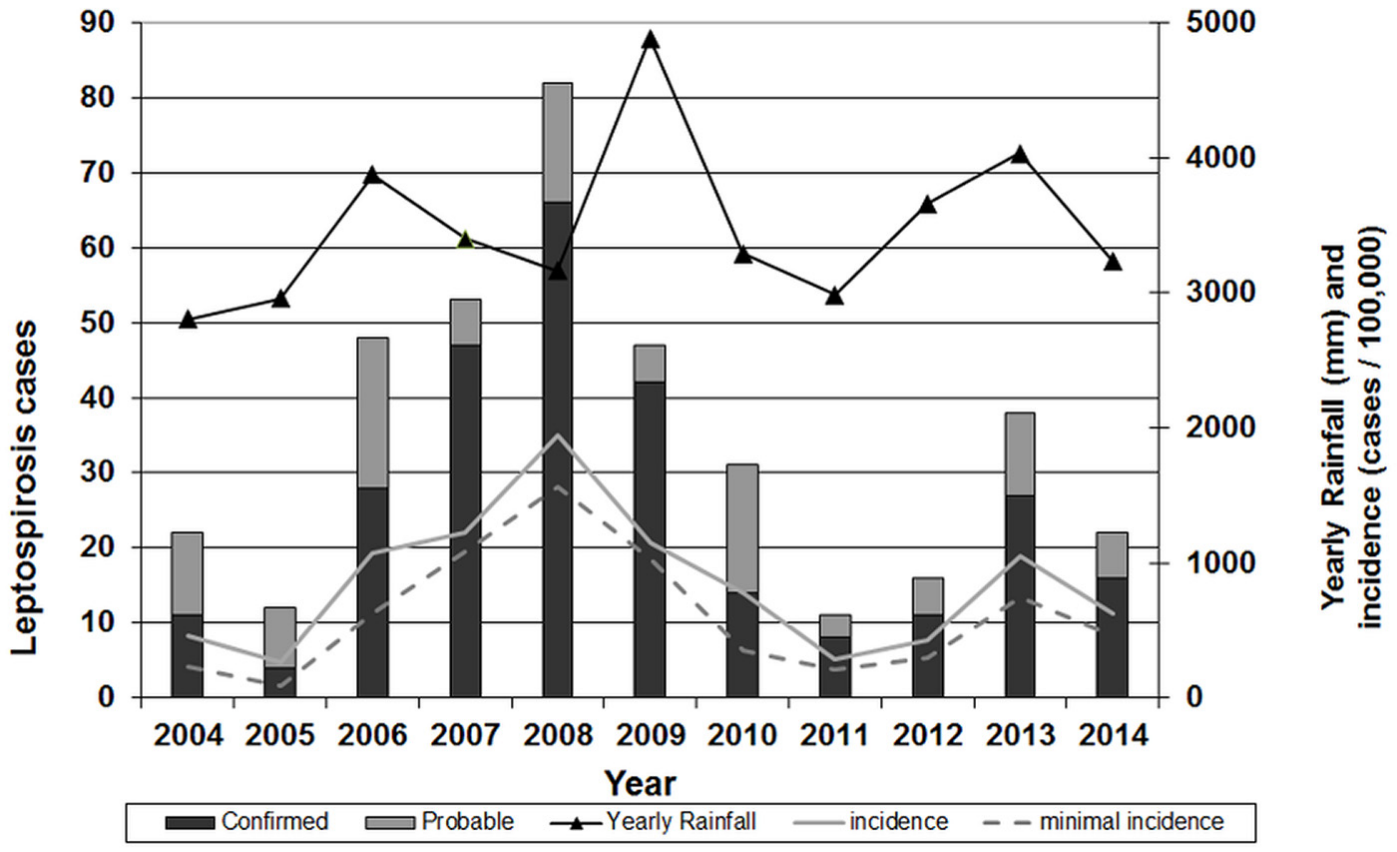
Number of probable and confirmed cases, incidence and total rainfall by year in Futuna. (Rainfall data kindly provided by Météo France). A minimal conservative value (“minimal incidence”) was calculated from only confirmed cases.

The annual incidence was 844 cases per 100,000 on average over the study period. It was notably highly variable from one year to another, notably increasing from 2005 to 2008 when a peak incidence of 1,945 cases per 100,000 inhabitants was observed. On average, the annual incidence over the 11-year study period was 844 cases per 100,000. If considering only confirmed cases, the mean incidence over the period was 745 cases per 100,000, with a peak value of 1,565 cases per 100,000 in year 2008. Interestingly, there was no significant relation between the total annual rainfall and the incidence (R² = 0.13) (Fig 3).

**Fig 4:**
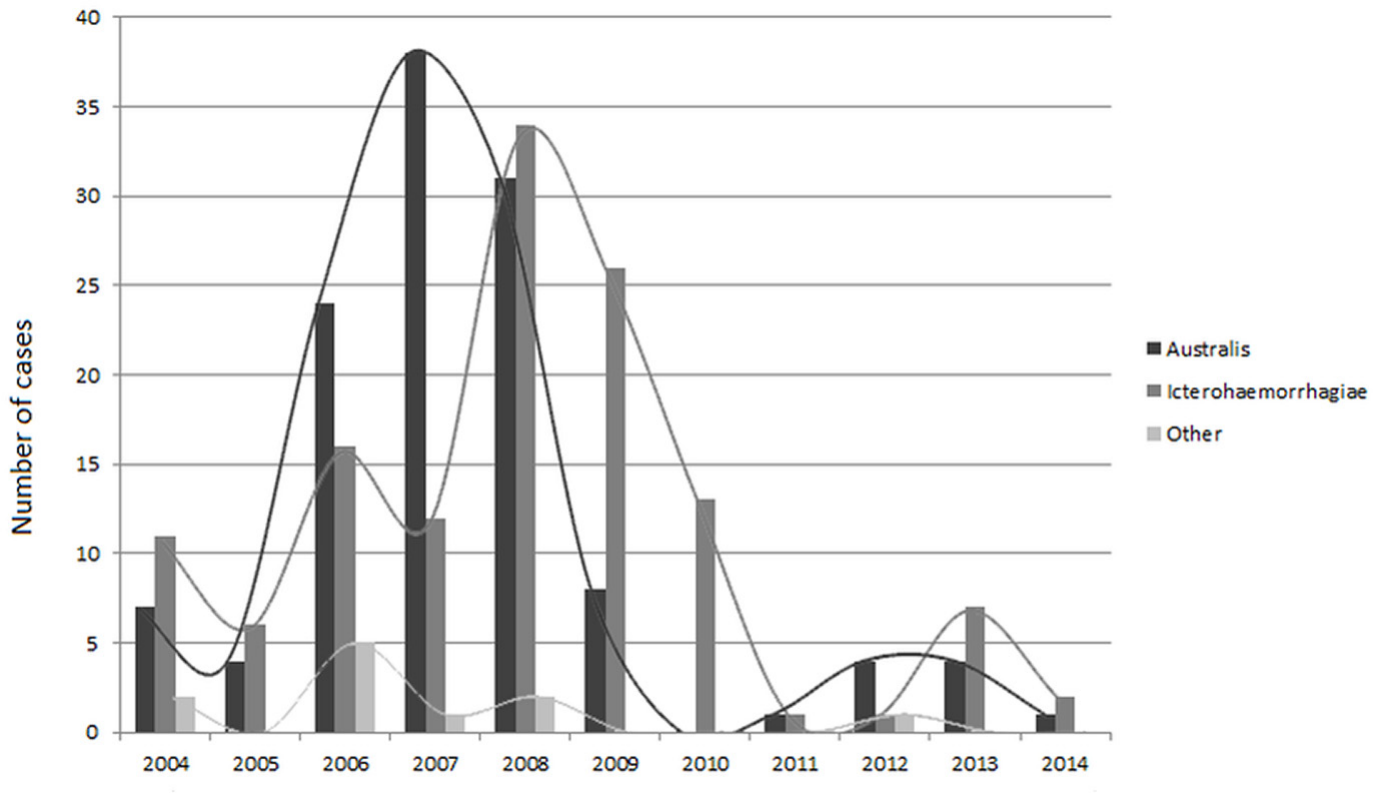
Number of cases by serogroup of infecting strain and year (N = 263).

The putative infecting serogroup could be identified for 263 out of the 382 cases. Serogroup Australis was highly dominant before 2008, Icterohaemorrhagiae was then largely dominant from 2008 onwards (Fischer exact test, p<0.001) as shown in Fig 4.

### Geographical pattern

**Fig 5:**
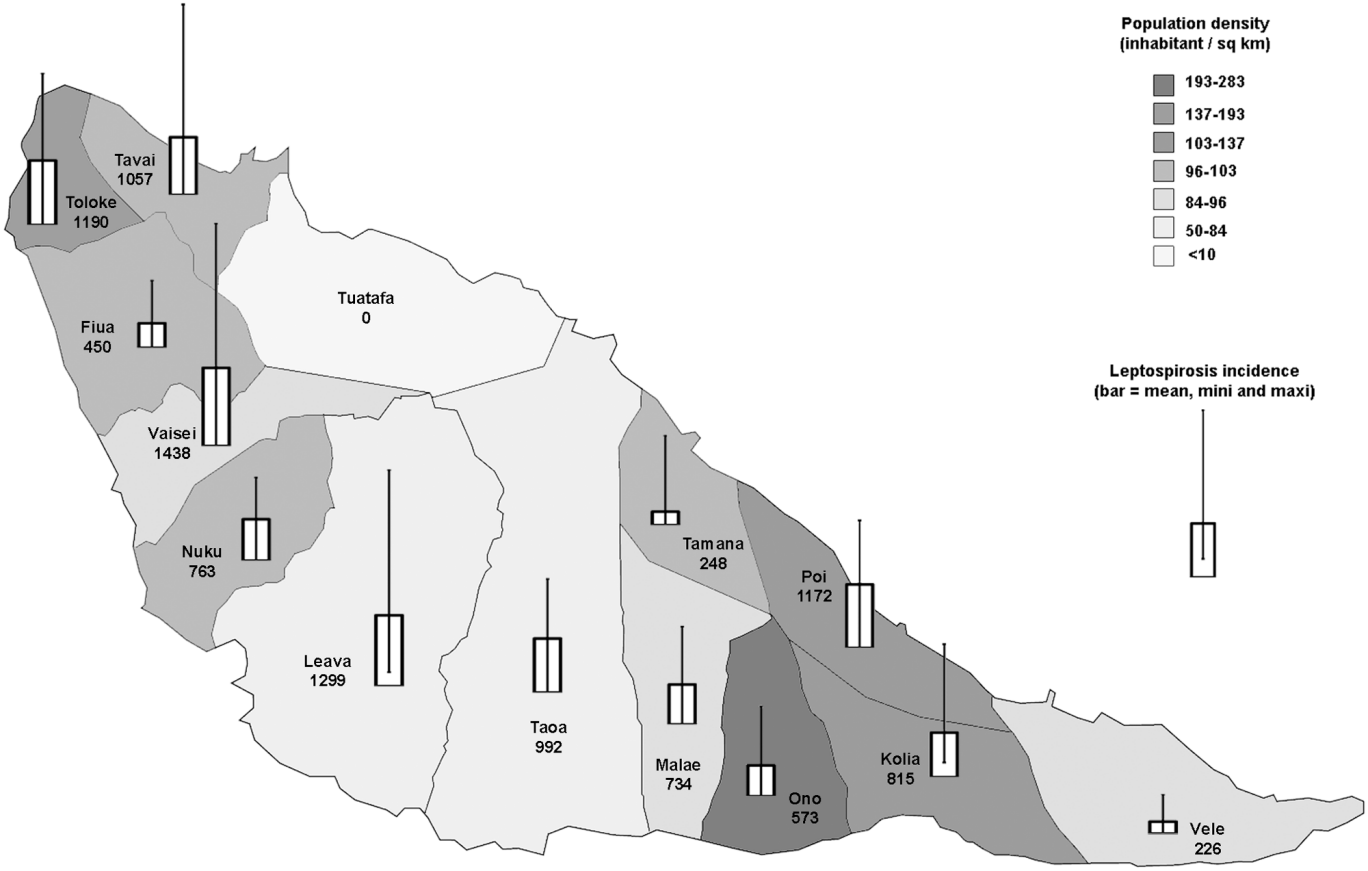
Map of Futuna Island with districts, population density and leptospirosis incidence over the period 2004–2014. “Mean, mini and maxi” indicate the average, minimum and maximum values for each district over the period.

There were cases in 13 out of 14 districts, as shown in Table 1 and Fig 5; the only district where no case was identified has a very low population. The mean annual incidence in affected districts ranged from 226 to 1391 per 100,000 inhabitants.

**Table 1:** Annual number of cases in each district, reference population (census 2008) and mean incidence over the period 2004–2014.

**District**	**2004**	**2005**	**2006**	**2007**	**2008**	**2009**	**2010**	**2011**	**2012**	**2013**	**2014**	**Total**	**Population (2008)**	**mean annual incidence**
**Fiua**	**0**	**0**	**1**	**4**	**4**	**3**	**0**	**1**	**1**	**2**	**0**	**16**	**323**	**450**
**Kolia**	**2**	**1**	**3**	**9**	**7**	**2**	**2**	**2**	**1**	**2**	**2**	**33**	**368**	**815**
**Laeva**	**2**	**1**	**9**	**3**	**15**	**5**	**6**	**1**	**2**	**7**	**3**	**54**	**378**	**1299**
**Malae**	**3**	**0**	**3**	**0**	**4**	**3**	**0**	**4**	**0**	**1**	**0**	**18**	**223**	**734**
**Nuku**	**0**	**0**	**1**	**2**	**4**	**3**	**2**	**2**	**2**	**4**	**2**	**22**	**262**	**763**
**Ono**	**3**	**0**	**3**	**7**	**11**	**4**	**7**	**0**	**0**	**6**	**1**	**42**	**666**	**573**
**Poi**	**6**	**2**	**5**	**5**	**4**	**5**	**2**	**0**	**0**	**2**	**2**	**33**	**256**	**1172**
**Tamana**	**0**	**0**	**0**	**0**	**3**	**0**	**0**	**0**	**1**	**1**	**0**	**5**	**183**	**248**
**Taoa**	**5**	**6**	**13**	**10**	**9**	**10**	**2**	**0**	**4**	**2**	**7**	**68**	**623**	**992**
**Tavai**	**0**	**2**	**2**	**3**	**6**	**0**	**3**	**1**	**0**	**3**	**0**	**20**	**172**	**1057**
**Toloke**	**0**	**0**	**5**	**6**	**5**	**7**	**4**	**0**	**3**	**2**	**1**	**33**	**252**	**1190**
**Tuatafa**	**0**	**0**	**0**	**0**	**0**	**0**	**0**	**0**	**0**	**0**	**0**	**0**	**34**	**0**
**Vaisei**	**1**	**0**	**3**	**2**	**8**	**5**	**3**	**0**	**2**	**4**	**3**	**31**	**196**	**1438**
**Vele**	**0**	**0**	**0**	**2**	**2**	**0**	**0**	**0**	**0**	**2**	**1**	**7**	**281**	**226**
**Total**	**22**	**12**	**48**	**53**	**82**	**47**	**31**	**11**	**16**	**38**	**13**	**382**	**4217**	**844**

Cluster analysis using SatScan identified several spatial clusters for 2004–2007 and 2010–2014, but none in 2008 or 2009, at the peak of incidence, as summarized in Table 2. At the beginning of the study period (2004–2008), the two separate districts of Poi and Taoa had a higher relative risk (RR) of 2.77 and 1.95 respectively. At the end of the period (2010–2014), another region made of 3 adjacent districts, namely Laeva, Nuku and Vaisei had a RR of 2.07. Over the complete period 2004–2014, people from the South-West region in districts of Taoa, Laeva, Nuku and Vaisei had a 1.48 higher RR of having leptospirosis (p = 0.002).

**Table 2:** Spatial clusters of leptospirosis identified through a spatial analysis.

Year	Total cases	Cluster	Cluster population	number of clustered cases	RR*	LLR†	p-value
**2004–2008**	135	Poi	190	18	2.77	6.32	0.01
		Taoa	531	34	1.95	5.05	0.03
**2008–2009**	129	No cluster					
**2010–2014**	118	Laeva-Nuku-Vaisei	784	43	2.07	6.64	0.004
**2004–2014**	382	Laeva-Nuku-Vaisei-Taoa	1315	175	1.48	7.08	0.002

*RR = relative risk.

^†^LLR = Log likelihood ratio.

## Discussion

From the first confirmed case in 1997 to 2003, the incidence of leptospirosis observed in Futuna by passive surveillance was 134 cases per 100,000 annually (data not shown). Active surveillance was implemented in 2004 and awareness of the medical community increased and maintained by the surveillance system. The annual incidence was on average 844 cases per 100,000 for the period 2004–2014 (Fig 3). This increase illustrates that active surveillance and improved awareness resulted in better identification of leptospirosis cases, as already shown in other settings [11, 12] and already recognized in the Asia-Pacific region [13]. However, even since this active surveillance was implemented, there has been a strong inter-annual variability in the incidence. The time series presented here strongly suggests a multi-year epidemic curve (Fig 3), with a peak incidence as high as 1,945 cases per 100,000 in 2008. High incidence of leptospirosis has already been reported from French Polynesia with 180 and 150 cases per 100,000 in Raiatea and Marquesas islands respectively [14], from the Seychelles with 101 cases per 100,000 [15] or up to 500 cases per 100,000 in particular hot spots in New Caledonia [16]. However, the mean annual incidence reported here was 844 cases per 100,000 over an 11-year period, and notably above 1,000 cases per 100,000 for 4 consecutive years (2006–2009) and even reached 1,945 cases per 100,000 in 2008. To the best of our knowledge, this is the highest incidence of leptospirosis ever reported. The use of very clear and restrictive case definitions together with high conservative cut-offs for serology (≥800 for MAT and above the recommendations of the manufacturer for ELISA) prevents from any overestimation. However, the high seroprevalence in hyper-endemic settings may lead to overestimating the real burden. Some of the positive serological results (either MAT or IgM ELISA when convalescent sera were unavailable) may only reflect earlier infections due to intense exposure to environmental contamination. Therefore, the case definition used discriminated between confirmed (i.e. ascertained by PCR, seroconversion or significant increase in serological titers) from probable (i.e. with a single positive serology) cases. When only confirmed cases were considered, the mean incidence over the period was 745 cases per 100,000, the incidence at the peak of the epidemic was still as high as 1081, 1,565 and 1,025 cases per 100,000 in 2007, 2008 and 2009 respectively. A recent study in Brazil suggested a 191:1 infection-to-disease ratio (95% CI, 82–542:1) [17]. Despite a different study setting, the incidence reported in 2006–2009 in Futuna together with this estimate highlight an omnipresent infection risk in Futuna during this period.

Based on careful consideration of this multi-year epidemic curve, there is evidence suggesting an ongoing epidemic caused by serogroup Australis (presumably maintained by pigs) and a starting epidemic caused by serogroup Icterohaemorrhagiae (known to be maintained by rats). Though these two outbreaks apparently peaked in 2007 and 2008 for Australis and Icterohaemorrhagiae respectively, the precise dynamics and the exact time shift cannot be ascertained, since they clearly overlap, largely contributing to this extremely high incidence in 2006–2009. The first period of our study (2004–2007) therefore suggests an outbreak related to swine farming, as already observed in other Pacific locations [18]. This also suggests that non-rodent *Leptospira* reservoirs can also cause a human outbreak. From 2008 onwards, serogroup Icterohaemorrhagiae became dominant, pointing to the more classical rodent reservoir. Whether changes in rat ecology and behavior in Futuna following the recent introduction of the black rat *Rattus rattus* has possibly contributed to this shift in leptospirosis epidemiology still remains unknown [19]. The second part of the study period showed a sharp decrease in the number of cases, with a minimum incidence of 285 cases per 100,000 in 2011, before a new increase in incidence was noted in 2013. Though this cyclical trend is probably mostly driven by ecological factors, it can be hypothesized that population immunity could also contribute, which would deserve consideration. Should population immunity actually be associated with this cyclical trend, it would suggest a protective population immunity of approximately 5-year duration. This data could be compared with possible other cyclical fluctuations in other settings with highly exposed populations.

Leptospirosis is known to be seasonal, and strongly associated with rainfall in tropical settings [1]. We also showed a correlation between incidence and rainfall two months earlier but our analysis used averaged data and should be refined using time-series analysis. The association between rainfall and leptospirosis was already studied and described in Réunion Island [20, 21], French Polynesia [22] Martinique [23] or Brazil [24], supporting the correlation observed in Futuna. Contrary to those findings, we found no link between total annual rainfall and incidence, suggesting that other factors contributed to this variability. The El Niño Southern Oscillation has its birthplace in the tropical Pacific Ocean. Whether it possibly contributes to the year-to-year variability in leptospirosis incidence in Futuna, as already demonstrated in New Caledonia [25] would also deserve consideration.

Within a hyper-endemic background, spatio-temporal clusters were found over short periods in particular districts (Poi and Taoa in 2004–2008 and a wider region including Laeva, Nuku and Vaisei since 2010). Though statistically significant, we were not able to explain the early 2004–2008 clusters. The more important cluster lasting for the total duration of the study in the Southwest region (Laeva, Nuku, Vaisei and Taoa) perhaps originates from an open rubbish dump in this region, known to provide a shelter for a large rat population.

Most cases were males (M/F = 5.4), in accordance with what is known for leptospirosis elsewhere. Whether it only reflects higher exposure of the male population through agricultural and farming activities or sex-specific susceptibility factors remains to be determined. The highest number of cases in the 10–19 y.o. age class should be considered, for being unusually a young age class for leptospirosis. Unfortunately, it was not possible to calculate age-specific incidence, because of the current massive emigration of young active people, making detailed demographics over an 11-year period very uncertain. However, based on the 2008 census, this highest number of cases does not correspond to highest age-specific incidence. A high number of cases in young age classes possibly reflects a frequent exposure of young active men involved in agriculture and farming activities. However, the hypothesis that older age classes are relatively protected by the immunity resulting from previous exposures also deserves consideration.

Most cases could be treated directly in Futuna where neither intensive care nor dialysis is available and the overall fatality rate was 2 / 382 (0.5%). This is in marked contrast with what was observed at the same time in New Caledonia, where patients frequently required admission in an intensive care unit and the fatality rate was higher with 3.7% [16]. The awareness of the medical community (leptospirosis to be considered as a first line diagnosis) and the general population (to rapidly seek medical advice in the event of a febrile illness) have possibly contributed to this low severity and fatality rates. The shift in serogroup discussed above was not significantly associated with an increase in clinical severity, as could have been expected with the dominance of Icterohaemorrhagiae [26].

## Conclusion

Leptospirosis is a major public health concern in Futuna with a very high incidence since more than a decade. In contrast, the sister Island of Wallis has a much lower incidence, despite similar cultural and socio-economical patterns, a comparison that would deserve further studies. A good awareness of medical doctors and the general population has most probably helped alleviating the burden of the disease through rapid antibiotic treatment. Prevention measures currently focus on encouraging the use of closed shoes or rubber boots for agriculture (mostly irrigated taro fields and pig farming in pens), but cultural habits will not change rapidly. Another concern is the open rubbish dump where rat populations proliferate; a community garbage management system would greatly contribute to control this particular problem.

## Supporting Information

S1 File MAT panel used in this study. (DOCX)

S1 STROBE Checklist (PDF)
