# Supplementary material for: An Unprecedented High Incidence of Leptospirosis in Futuna, South Pacific, 2004 – 2014, Evidenced by Retrospective Analysis of Surveillance Data
Source: PLoS One. 2015 Nov 3;10(11):e0142063. doi: 10.1371/journal.pone.0142063 (PMC4631516; doi:10.1371/journal.pone.0142063)
Supplement: S1 File — (DOCX) [file pone.0142063.s001.docx]

MAT Panel used at Institut Pasteur in New Caledonia

| Species | Serogroup | Serovar | Strain |
| --- | --- | --- | --- |
| *L. interrogans* | Australis | Australis | Ballico |
| *L. interrogans* | Autumnalis | Autumnalis | Akiyami A |
| *L. borgpetersenii* | Ballum | Ballum | Castellon 3 |
| *L. interrogans* | Bataviae | Bataviae | Van Tienen |
| *L. interrogans* | Canicola | Canicola | Hond Utrecht |
| *L. interrogans* | Icterohaemorrhagiae | Icterohaemorrhagiae | Verdun |
| *L. interrogans* | Icterohaemorrhagiae | Copenhagenii | Winjberg |
| *L. noguchii* | Panama | Panama | CZ 214 K |
| *L. interrogans* | Pomona | Pomona | Pomona |
| *L. interrogans* | Pyrogenes | Pyrogenes | Salinem |
| *L. borgpetersenii* | Tarassovi | Tarassovi | Mitis Johnson |
| *L. biflexa* | Semaranga | Patoc | Patoc I |
